# Pifithrin-α alters p53 post-translational modifications pattern and differentially inhibits p53 target genes

**DOI:** 10.1038/s41598-020-58051-1

**Published:** 2020-01-23

**Authors:** Jiawei Zhu, Madhurendra Singh, Galina Selivanova, Sylvain Peuget

**Affiliations:** Department of Microbiology, Tumor and Cell Biology, Karolinska Institutet, Stockholm, Sweden

**Keywords:** Tumour-suppressor proteins, Small molecules

## Abstract

Pifithrin-α (PFT-α) is a small molecule which has been widely used as a specific inhibitor of p53 transcription activity. However, its molecular mechanism of action remains unclear. PFT-α has also been described to display potent p53-independent activity in cells. In this study, we addressed the mechanism of action of PFT-α. We found that PFT-α failed to prevent the effects of Mdm2 inhibitor Nutlin-3 on cell cycle and apoptosis in several cancer cell lines. However, PFT-α rescued normal primary fibroblasts from growth inhibition by Nutlin-3. PFT-α displayed a very limited effect on p53-dependent transcription upon its activation by Nutlin-3. Moreover, PFT-α inhibitory effect on transcription was highly dependent on the nature of the p53 target gene. PFT-α attenuated post-translational modifications of p53 without affecting total p53 protein level. Finally, we found that PFT-α can decrease the level of intracellular reactive oxygen species through activation of an aryl hydrocarbon receptor (AHR)-Nrf2 axis in a p53-independent manner. In conclusion, PFT-α inhibits only some aspects of p53 function, therefore it should be used with extreme caution to study p53-dependent processes.

## Introduction

p53 (*TP53*), the guardian of the genome, is a tumor suppressor mutated in more than 50% of human cancers^1^. Wild-type p53 usually remains at low level in cells because of constant Mdm2-mediated proteasome-dependent degradation^2^. In response to various stresses, such as DNA damage, p53 is stabilized and exerts its transcription factor activity by binding on the promoter of its target genes. p53 regulates a range of biological functions, including apoptosis, cell cycle and DNA repair. Pharmacological reactivation of p53 is a promising approach to treat cancer patients, and several compounds designed to reactivate p53 are undergoing clinical trials. However, p53 activation during conventional chemotherapy or irradiation also leads to severe side effects in healthy tissues^3^. Therefore, several p53 inhibitors have been developed in an attempt to reduce the side effects of cancer therapy.

The small molecule PFT-α has been identified as a specific p53 inhibitor using p53-dependent LacZ/β-Gal reporter assay for screening of chemical library^4^. PFT-α has been shown to protect mice from the harmful effects of gamma irradiation^4^, to protect mouse neurons from death^5^ and to enhance the recovery of sub-ventricular zone of mice after brain stroke^6^. The exact mechanism of how PFT-α suppresses p53 activity is still elusive. It has been first described to inhibit p53 transcriptional activity by decreasing its protein level in ConA cells^4^, but to inhibit p53 DNA binding activity through an unknown mechanism without affecting its protein level in neurons^4^. PFT-α has been described to prevent the nuclear translocation of p53 by direct interaction with hsp90^7^. Surprisingly, PFT-α has been shown to enhance topotecan-induced cell death in MCF7, BGC823 and HepG2 cells in a p53-dependent way^8^. Moreover, accumulating evidence indicates that PFT-α could prevent cell apoptosis in a p53-independent manner, via inhibition of caspase 3 and 9 activation, via inhibition of Rb hyper-phosphorylation through cyclin-D1^9^, or via activation of macro-autophagy^10^. PFT-α has also been described to activate the aryl hydrocarbon receptor (AHR) pathway independently of p53^11^.

Despite its unclear molecular mechanism and potential off-target effects, PFT-α is still widely used as a specific p53 inhibitor to investigate p53-dependent response, for instance, in autophagy^12^, response to drugs^13^, DNA damage^14^, neurogenesis and angiogenesis^15^ or cardiac hypertrophy^16^. As activation of the p53 pathway was recently described to inhibit the CRISPR/Cas system^17,18^, PFT-α was also used in attempts to increase the efficiency of genome editing^19^. In our study, we found that PFT-α did not protect cells from p53 reactivation in different models and had only a mild effect on p53 transcriptional activity. Moreover, the effect of PFT-α was highly dependent on the nature of the p53 target gene. Interestingly, we show for the first time that PFT-α can impair p53 post-translational modifications (PTMs). As we also show that PFT-α can exert an antioxidant effect through the AHR/Nrf2 pathway independently of p53, our study suggests that its inhibition of p53 transcriptional activity may be due to alterations of p53 PTMs and therefore is highly context- and gene-dependent.

## Results

### PFT-α fails to protect cancer cells, but not normal cells from p53-mediated growth suppression

**Figure 1:**
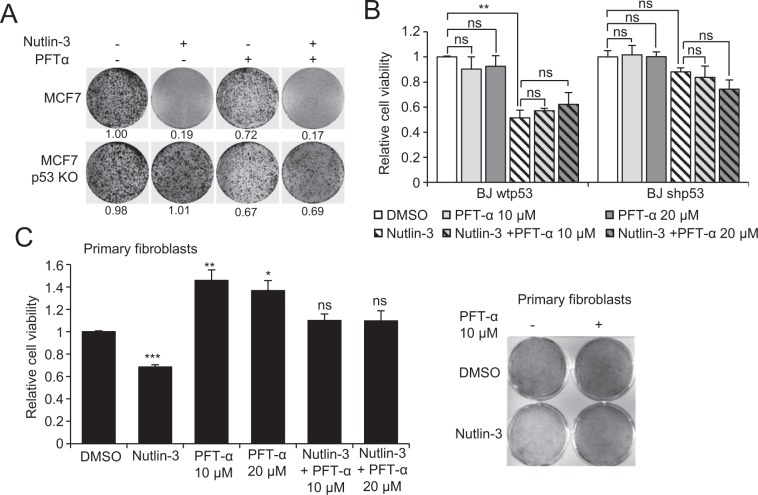
Effect of PFT-α on Nutlin-3-induced growth suppression. (**A**) Crystal violet staining of MCF7 and MCF7 p53KO cells after 10 µM Nutlin-3 with or without PFT-α (20 µM) treatment for 72 h. Relative quantification is indicated below the pictures. (**B**) Resazurin cell viability assay of BJ fibroblasts (wtp53 and shp53) upon 10 µM Nutlin-3 and PFT-α treatment for 72 h. The values are reported as relative cell viability normalized to DMSO treatment group and represent the mean ± SD of three replicates. (**C**) Resazurin cell viability assay and crystal violet staining of primary fibroblasts with Nutlin-3 (10 µM) and PFT-α treatment for 72 h. The values are reported as relative cell viability normalized to DMSO treatment group and represent the mean ± SD of three replicates.

To investigate the efficacy of PFT-α inhibition of p53 in cancer cells, we reactivated p53 by Nutlin-3, which inhibits the E3-ubiquitin ligase MDM2 by direct binding, disrupts the p53-MDM2 complex and prevents subsequent p53 degradation by the proteasome^20^. As expected, the growth of wild type (wt) p53 MCF7 breast carcinoma cells was suppressed by Nutlin-3 treatment, whereas it had no effect in p53-depleted (KO) cells, in line with the notion that Nutlin-3 induces cell cycle arrest and/or apoptosis specifically through p53 activation. However, PFT-α treatment did not protect cells from Nutlin-3-mediated growth suppression in MCF7, nor in A375 cells (Fig. 1A and Supplementary Fig. S1A–C). Moreover, we observed a slight but significant cytotoxic effect upon 20 µM PFT-α treatment of MCF7 p53wt cells (Supplementary Fig. S1B).

To check if PFT-α could inhibit p53-mediated cell cycle arrest in normal cells, we treated wt and p53-depleted (KD) immortalized human fibroblasts BJ with Nutlin-3 in the presence or absence of PFT-α. Nutlin-3 is described to only induce cell cycle arrest but not apoptosis in fibroblasts^21^. Again, our results show that Nutlin-3 induces p53 dependent growth suppression in BJ fibroblasts which cannot be rescued by PFT-α (Fig. 1B). Then, we tested the effect of PFT-α in primary, non-immortalized normal fibroblasts. Interestingly, PFT-α *per se* was promoting primary fibroblasts growth, which compensates Nutlin-3-induced growth suppression in crystal violet and resazurin assays (Fig. 1C). It has been described that another pifithrin compound, pifithrin-μ (PFT-μ) but not PFT-α can protect cells from Nutlin-3-induced killing^22^. Therefore, we also tested the effect of PFT-μ in MCF7 and A375. In our models, PFT-μ as well did not inhibit p53-induced growth suppression (Supplementary Fig. S1B).

### PFT-α has differential inhibitory effect on p53 transcriptional targets

**Figure 2:**
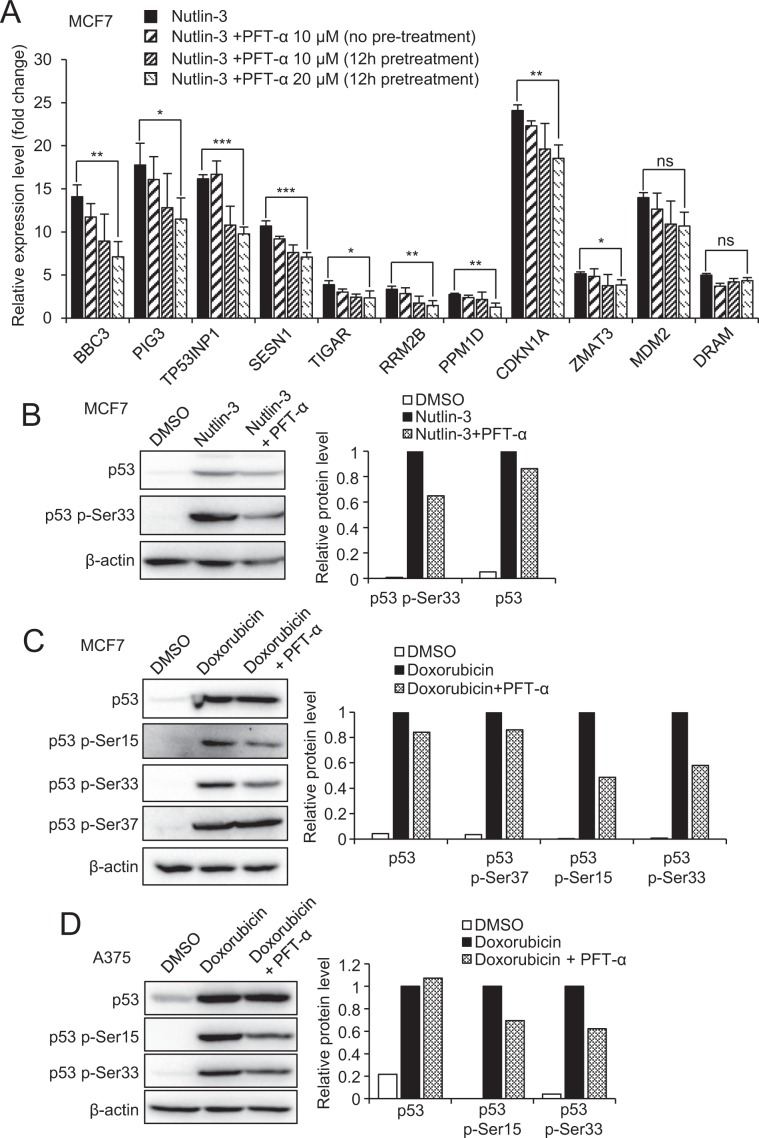
Effect of PFT-α on p53 transcriptional target genes and p53 PTMs. (**A**) qPCR for the detection of mRNA level of p53 transcriptional target genes in MCF7 cells upon Nutlin-3 (10 µM) with or without 12 h pre-treatment with 20 µM PFT-α. The values are reported as fold change relative to DMSO treatment group and represent the mean ± SD of three independent experiments performed in three replicates. (**B**) Western blot to detect the protein level of p53 and p53 p-Ser33 upon 8 h Nutlin-3 treatment (10 µM) with or without PFT-α (20 µM, 12 h pre-treatment) in MCF7 cells. Densitometric analysis of the bands was performed using ImageJ software, the ratio of p53, p53 p-Ser33/β-actin for DMSO, Nutlin-3 and Nutlin-3 plus PFT-α treatment was quantified and then normalized with Nutlin-3 treatment group. (**C**) Western blot to detect the protein level of p53, p53 p-Ser33 and p53 p-Ser15 upon 8 h doxorubicin treatment (1 µM) with or without PFT-α (20 µM, 12 h pre-treatment) in MCF7 cells. Densitometric analysis of the bands was performed using ImageJ software, the ratio of p53 p-Ser33/β-actin and p53 p-Ser15/β-actin for DMSO, doxorubicin and doxorubicin plus PFT-α treatment was quantified and then normalized with doxorubicin treatment group. (**D**) Same experiment as in C, performed in A375 cells.

To investigate the effects of PFT-α on p53 transcriptional activity upon Nutlin-3, we treated MCF7 cells with PFT-α at several conditions described to inhibit p53 transcription in the literature, without having strong cytotoxicity^23,24^ (10 µM and 20 µM, with or without 12 h pre-treatment). The inhibitory effect of PFT-α on p53 target genes was negligible, and only detectable upon pre-treatment for 12 h prior to Nutlin-3 treatment (Fig. 2A). Even in this condition, PFT-α cannot protect cells from p53 activation-mediated growth suppression, neither from cell cycle arrest in MCF7 cells^25^ nor from apoptosis in A375 cells^26^ (Supplementary Fig. S2A).

We then investigated the effect of PFT-α on several well characterized p53 target genes involved in a variety of cell responses. We confirmed the p53-dependency of these genes in response to Nutlin-3 using MCF7 p53wt and p53KO cells (Supplementary Fig. S2B). Interestingly, we observed that PFT-α had a drastically different inhibitory effect on different p53 target genes (Fig. 2A). Our data show that *BBC3* (PUMA), *PIG3, TP53INP1*, *RRM2B, PPM1D* (WIP1), *SESN1* and *TIGAR* induction upon Nutlin-3 was moderately inhibited (decreased by 35% to 50%) by PFT-α (20 µM, 12 h pre-treatment condition), while the effect on the transcription of *ZMAT3* and *CDKN1A* (p21) was limited (induction decreased by only 23% and 25% respectively). Moreover, no significant transcriptional inhibition was observed for *MDM2* and *DRAM*. We also confirmed by western blot that MDM2 and p21 were not inhibited at protein level by PFT-α upon Nutlin-3-induced p53 stabilization (Supplementary Fig. S2C).

### PFT-α impairs p53 post-translational modifications

p53 is tightly regulated by diverse PTMs, such as phosphorylation on its different serine residues, which influence its promoter selectivity and the induction of its target genes^27^. Therefore, we hypothesized that PFT-α could affect PTMs of p53, hence selectively inhibiting the activation of specific p53 target genes. We assessed the level of several phosphorylated forms of p53 by western blot using specific antibodies. We observed that phosphorylation of p53 on Ser33 upon Nutin-3 treatment was efficiently inhibited by PFT-α (Fig. 2B). Upon p53 activation by doxorubicin treatment in several cancer cell lines, PFT-α efficiently inhibited both Ser33 and Ser15 phosphorylation, but not Ser37 (Fig. 2C,D and Supplementary Fig. S2D). Our data demonstrate that PFT-α can affect specific p53 PTMs, which could lead to differential expression of p53 target genes.

### PFT-α decreases reactive oxygen species (ROS) level through an AHR/Nrf2 axis independently of p53

**Figure 3:**
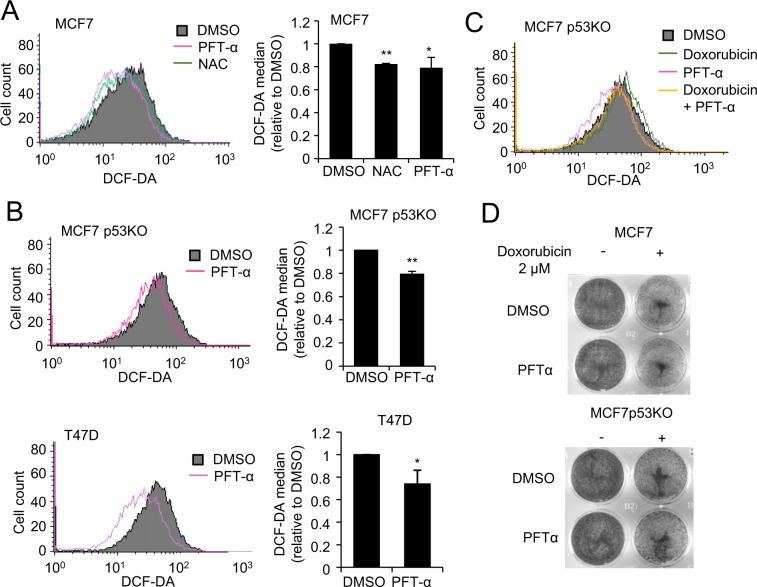
Antioxidant activity induced by PFT-α. (**A**) DCF-DA staining of ROS levels upon NAC (10 mM) and PFT-α (20 µM) treatment for 20 h in MCF7 cells (left panel), detected using flow cytometry. For quantification of ROS levels (right panel), the values are reported as percentage relative to DMSO treatment group and represent the mean ± SD of three independent experiments. (**B**) DCF-DA staining for ROS levels upon 20 h PFT-α treatment (20 µM) in MCF7 p53KO cells (upper left panel) and T47D cells (lower left panel). For quantification of ROS levels (right panels), the values are reported as percentage relative to DMSO treatment group and represent the mean ± SD of three independent experiments. (**C**) DCF-DA staining for ROS levels upon 8 h Doxorubicin (1 µM) with or without addition of PFT-α (20 µM, 12 h pre-treatment) in MCF7 p53KO cells. (**D**) Crystal violet staining to detect cell viability upon 48 h Doxorubicin treatment (1 µM)) in the presence or absence of PFT-α (20 µM) in both MCF7 and MCF7 p53KO cells.

As redox homeostasis has been described to be one of the factors regulating p53 phosphorylation and determining cell fate^28,29^, we investigated the effects of PFT-α on intracellular ROS by monitoring 2′,7′-Dichlorofluorescin diacetate (DCF-DA) staining by flow cytometry. Our results show that PFT-α treatment leads to a significant decrease of intracellular ROS level in MCF7 cells, to the same extent as the well-known antioxidant N-Acetylcysteine (NAC) (Fig. 3A). Moreover, the antioxidant effect of PFT-α is p53-independent, as a similar response was observed in p53 KO MCF7 cells, in p53 mutant T47D cells, and to a lesser extend in A375 p53 KO cells (Fig. 3B and Supplementary Fig. S3A). PFT-α significantly decreased doxorubicin-induced ROS formation, but failed to rescue doxorubicin-induced cell death (Fig. 3C,D), which is in agreement with previous work showing that doxorubicin-induced cell death is independent of ROS^30^.

**Figure 4:**
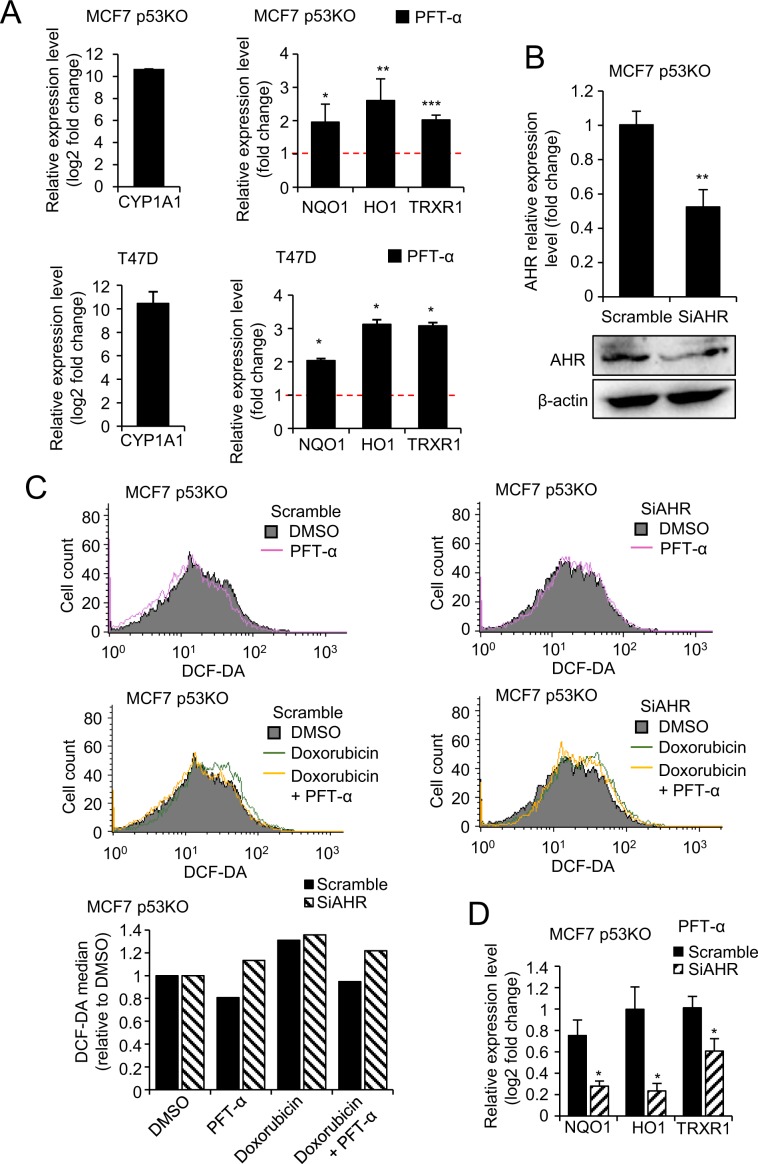
Activation of AHR/Nrf2 pathway by PFT-α. (**A**) qPCR to detect mRNA level of *CYP1A1, NQO1*, *HO1* and *TRXR1* upon 20 h PFT-α treatment (20 µM) in MCF7 p53KO cells (upper part) and T47D cells (lower part). Relative expression level of *CYP1A1* is shown in log2 scale; relative expression level of *NQO1*, *HO1* and *TRXR1* are shown as fold change, both normalized with DMSO treatment. All values represent the mean ± SD of two times independent experiments performed in three replicates. (**B**) Knock-down efficiency of *AHR* siRNA as detected by qPCR (upper part) and western blot (lower part). Relative expression level is shown as fold change normalized to scramble siRNA. All values represent the mean ± SD of two independent experiments performed in three replicates. (**C**) DCF-DA staining of ROS levels upon doxorubicin treatment (1 µM, 8 h) with or without PFT-α (20 µM, 12 h pre-treatment) in MCF7 p53KO cells transfected with scramble siRNA and siAHR. For quantification of ROS levels (lower panel), the values are reported as percentage relative to DMSO treatment group. (**D**) qPCR to detect mRNA level of Nrf2 targets *NQO1*, *HO1* and *TRXR1* upon 20 h PFT-α treatment (20 µM) in MCF7 p53KO cells transfected with scramble siRNA and siAHR.

Interestingly, PFT-α was previously shown to be a potent agonist of AHR^11^. AHR activates Nrf2, the master switch of cell antioxidant response^31^. Therefore, we addressed the question whether PFT-α could decrease intracellular ROS by activating the Nrf2 pathway through AHR. PFT-α treatment dramatically induced the expression of one of the main AHR targets, the cytochrome P450 (CYP1A1) in p53 KO MCF7 cells, confirming its activation of AHR independently of p53. Then we investigated the effect of PFT-α on Nrf2 in MCF7 p53KO cells. Nrf2 pathway was activated by PFT-α treatment, as the mRNA level of Nrf2 targets *NQO1, HO1, and TRXR1* was significantly upregulated (Fig. 4A and Supplementary Fig. S3B). To confirm the involvement of the AHR/Nrf2 pathway, we performed siRNA-mediated silencing of AHR (Fig. 4B), which almost completely reversed the antioxidant effect of PFT-α alone, as well as its ability to prevent ROS formation upon doxorubicin treatment (Fig. 4C). Accordingly, activation of Nrf2 pathway by PFT-α was partially inhibited upon AHR silencing (Fig. 4D). Moreover, H1299 lung carcinoma cells, which express low levels of AHR^32^, are not responsive to PFT-α in terms of ROS decrease or Nrf2 pathway activation, consistent with our data (Supplementary Fig S3C–E).

**Figure 5:**
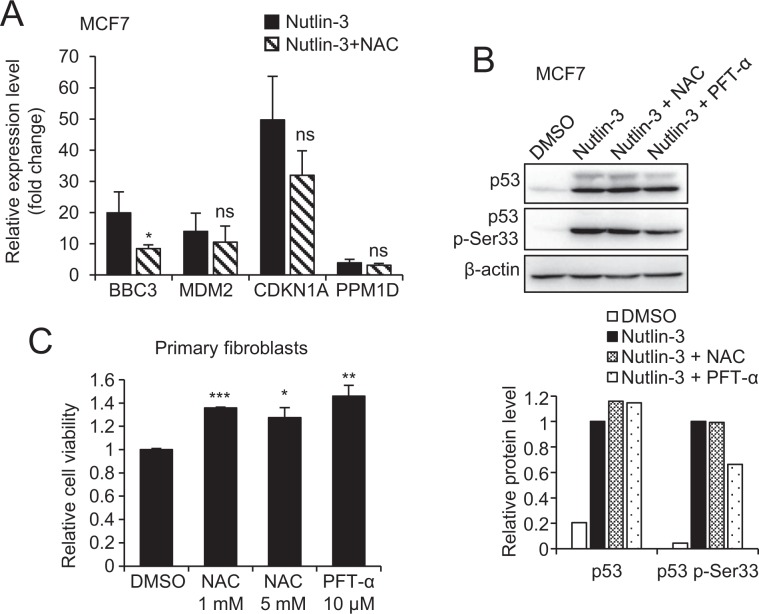
Comparison of PFT-α and NAC on p53 PTMs. (**A**) qPCR for mRNA levels of BBC3, CDKN1A, MDM2 and PPM1D upon 8 h Nutlin-3 treatment (10 µM) in the presence of NAC (10 mM, 12 h pre-treatment) in MCF7 cells. Relative expression level is reported as fold change relative to DMSO treatment and represents the mean ± SD of three replicates. (**B**) Western blot to detect the protein level of p53 and p53 p-Ser33 upon 8 h Nutlin-3 treatment (10 µM) in MCF7 cells in the presence of PFT-α (20 µM, 12 h pre-treatment) or NAC (10 mM, 12 h pre-treatment). Densitometric analysis of the bands was performed using ImageJ software, the ratio of p53, p53 p-Ser33/β-actin is normalized to Nutlin-3 treatment group. (**C**) Resazurin cell viability assay of primary fibroblasts upon NAC or PFT-α treatment for 72 h. The values are reported as relative cell viability normalized to DMSO treatment group and represent the mean ± SD of three replicates.

Therefore, we hypothesized that PFT-α effects on p53 PTMs could be caused by its antioxidant potential. First, we confirmed that antioxidants such as NAC can also inhibit p53 transcriptional activity on some of its target genes. Our results show that NAC significantly inhibited the induction of *BBC3* upon Nutlin-3 treatment (Fig. 5A), but none of the other p53 target genes investigated. We then compared the effect of PFT-α and NAC on p53 phospho-Ser33 upon Nutlin-3 treatment. While PFT-α robustly decreased the levels of p-Ser33, in contrast NAC did not inhibit p53 phosphorylation (Fig. 5B). Thus, while PFT-α exhibits intracellular antioxidant effect through activation of AHR/Nrf2 pathway, this cannot serve as a mechanism of PFT-α-mediated impairment of p53 PTMs. However, NAC exerted a similar effect as PFT-α on promoting primary fibroblasts growth, suggesting that this phenotype is related to its antioxidant properties (Fig. 5C**)**.

## Discussion

PFT-α, albeit originally designed to attenuate p53-dependent toxic side effects of anti-cancer therapy, is widely used in research to study p53-dependency of cellular processes. However, accumulating data suggest that PFT-α is not very specific for p53. Moreover, it is not very efficient at inhibiting p53^10,33,34^. In this study, we found that PFT-α cannot prevent p53-dependent effects in several models of p53 reactivation. Moreover, its ability to inhibit p53 transcription is restricted to only some of p53 target genes, indicating a more complex effect than inhibition of p53 transcriptional activity. Our data suggest that PFT-α alters the PTMs profile of p53, hence affecting its transcription activity on different targets.

p53 is heavily modified by PTMs, such as phosphorylation, acetylation, ubiquitination and others, and p53-mediated cellular functions are highly orchestrated via the intermingle of these diverse events^29^. It is hypothesized that the profile of p53 PTMs induced by a specific stimulus forms a “barcode” which results in a specific cellular response^35^. For example, phosphorylation of p53 on Ser46 was found to induce apoptosis but not cell-cycle arrest^36^. p53 PTMs are therefore dependent on the type of p53-reactivating signals and vary depending on the intracellular context, such as DNA damage or oxidative stress.

However, even though we have shown that PFT-α can decrease intracellular ROS, we could not link the decreased ROS level to the observed changes in p53 PTMs. Therefore, the mechanism of PFT-α – exerted effect on p53 PTMs remains to be elucidated. We speculate that PFT-α could block p53 PTMs either by direct binding or by affecting the molecular machinery that modifies p53. p53 can be phosphorylated by multiple kinases that have little specificity for a specific residue, each kinase phosphorylating several residues and each residue phosphorylated by several kinases^37^. So far, more than 10 different kinases have been described, including DYRK2, CHK1, CHK2, CK2, HIPK2, JNK, LRRK2, p38, PKCδ, and PLK3. Interestingly, a study showed that PFT-α displays effects similar to p38MAPK inhibitors on the regulation of embryonic stem cells differentiation^38^, and p38 kinase can phosphorylate p53 on Ser33, Ser15 and Ser37^39^. Therefore, it is possible that PFT-α could inhibit PTMs of p53 by inhibiting p38/MAPK signaling. However, we did not observe any inhibition of phosphorylated MAPK, ERK1 and p38α upon PFT-α treatment (data not shown).

Our data suggest that p53 is probably not the main target of PFT-α in cells. AHR is strongly activated upon PFT-α treatment, and is known to regulate a number of cellular processes beside Nrf2, through transcriptional activation, modulation of protein interactions or epigenetic mechanisms^40^. Therefore, AHR activation could trigger a chain of secondary signaling events leading to the inhibition of p53 transcriptional activity, but we cannot rule out the possibility that PFT-α has additional targets. Further studies are therefore needed to identify which proteins are bound and inhibited. However identifying PFT-α direct target(s) remains challenging, and will probably require broad-scales approaches such as genome-wide CRISPR-Cas KO screens^41^ or thermal proteome profiling^42^.

In our experiments, PFT-α cannot protect cells from apoptosis and cell cycle arrest upon p53 activation, in agreement with previous study^33^. Interestingly, PFT-α *per se* promotes cell growth in primary fibroblasts but causes a slight inhibition of cancer cell proliferation. PFT-α has previously been shown to enhance neural progenitor cells and rat hepatocytes proliferation, however, these effects were linked to p53 inhibition and decreased p21 expression^6,43^. As NAC has a similar to PFT-α effect on cell proliferation in primary fibroblasts, this phenotype could also be linked to the activation of antioxidant machinery by PFT-α. Hence, growth promotion by PFT-α could compensate the cell cycle arrest induced by Nutlin-3 treatment in primary fibroblasts. On the other hand, PFT-α showed slight inhibition of cancer cells proliferation at the same doses. This finding suggests that PFT-α could be interesting to use in combination with p53-reactivating anticancer therapies in the future.

In conclusion, the efficiency of PFT-α as specific p53 inhibitor appears to be highly questionable and context dependent. In our models, it did not inhibit p53-dependent cell growth suppression and had target gene- and model-dependent effects on p53 transcription, probably due to indirect inhibition of p53 PTMs. In addition, PFT-α has notable p53-independent effects such as strong induction of the AHR pathway. However, its differential properties in normal primary cells and cancer cells may have interesting clinical potential in combination with p53-reactivating compounds.

## Methods

### Cell lines and compounds

Human breast cancer cell lines MCF7 (p53 wt) and T47D (p53 R248Q mutant), colon cancer cell line HCT116 (p53 wt), melanoma cancer cell line A375 (p53 wt), lung carcinoma cell line H1299 (p53 null) and immortalized human fibroblast cell line BJ were maintained in DMEM medium supplemented with 10% FBS (Gibco). Human primary fibroblasts were maintained in DMEM/F12 medium supplemented with 10% FBS (Gibco). CRISPR/Cas9-mediated p53 deletion was performed in MCF7 and A375 (Supplementary Fig. S1A). Briefly, stable Cas9 expressing cells were established and then transfected two times with p53 sgRNA. Enrichment of p53-deleted cells was performed by Nutlin-3 treatment (10 μM) for 3 days. Cas9 expressing vector and px330 vector coding for sgRNA targeting p53 exon 4 were kindly provided by Vera Grinkevich (Welcome Trust Sanger Institute, Cambridge, United Kingdom). h-Tert immortalized BJ fibroblasts and its p53-depleted by shRNA derivative line were a kind gift from Reuven Agami, The Netherlands. PFT-α, PFT-µ, Nutlin-3 and NAC were purchased from Sigma-Aldrich. Doxorubicin was purchased from Selleckchem. Except when stated otherwise, cells were treated with 20 µM PFT-α, 10 µM Nutlin-3, 10 mM NAC and 1 µM Doxorubicin for the indicated time.

### Cell viability assays

For crystal violet staining experiments, cells were seeded in 12-well plates the day before the experiment (0.2 million cells per well), then pre-treated and treated as indicated (12 h NAC or PFT-α pre-treatment followed by 48 h Nutlin-3 treatment). Then, cells were washed in PBS, fixed for 15 min with ice-cold 70% ethanol, and stained with 0.2% crystal violet. Quantification was performed using ImageJ software.

For resazurin assay, cells were seeded in 96 wells plates (10.000 cells/well). The day after, cells were treated as indicated for 72 h and incubated 2 h with 5 μM resazurin (Sigma-Aldrich) before fluorescence measurement on a VICTOR X5 plate reader (PerkinElmer).

### Real time quantitative PCR assay

For qPCR experiments, cells were seeded in 6 well plates (0.4 million cells per well) and treated the day after for the indicated time. Then, the cells were harvested, and total RNA was extracted using Aurum total RNA mini kit (Bio-Rad) according to supplier instructions. 1 µg of total RNA was reverse-transcribed using iScript cDNA synthesis kit (Bio-Rad). mRNA quantification was performed using a fluorescence-based real-time RT-PCR technology (Sso Advanced Universal SYBRGreen SuperMix, Bio-Rad). RPL13A was used as housekeeping gene. Error bars represent standard deviation from mean of at least three independent experiments. The sequences of qPCR primers used are detailed in Supplementary Table 1.

### Western blots

Cell were treated as for qPCR experiments. To prepare protein lysates, cells were harvested, washed, and lysed in ice cold RIPA buffer (150 mM NaCl, 5 mM Tris [pH 8.0], 1% Triton X-100, 0.5% sodium deoxycholate, 0.1% SDS) supplemented with complete protease inhibitor cocktail (Roche) and PhosSTOP phosphatase inhibitors (Roche). Western blotting was performed according to standard protocols. Specific antibodies against AHR (NB100-2289, Novus Biologicals) MDM2 (33–7100, ThermoFisher Scientific), p21 (610233, BD Transduction), p53 (sc-126, Santa Cruz Biotechnology), p53 p-Ser33 (2526 s, Cell Signaling), p53 p-Ser15 (9286 S, Cell Signaling), p53 p-Ser37 (9289 S, Cell Signaling) were used for endogenous protein detection. As loading control, anti-β-actin (MAB1501, Millipore) or anti-GAPDH (sc-365062, Santa Cruz Biotechnology) was used after stripping the membrane with Restore Plus Stripping Buffer (ThermoFisher Scientific). Densitometric analysis of the bands was performed using ImageJ software (http://imagej.nih.gov/ij/). Full-length uncropped blots are displayed in Supplementary Fig. S4.

### DCF-DA reactive oxygen species detection

For ROS measurement experiments, 0.4 million cells (0.2 million cells in the case of 12 h pre-treatment of NAC or PFT-α) were seeded in 6 well plates. The day after, doxorubicin was added for 8 h treatment, then dishes were washed and incubated 30 min with 10 μM DCF-DA in serum and antibiotics free medium. Then cells were trypsinized, washed twice with PBS and fluorescence was analyzed by a FACSCalibur flow cytometer (BD Biosciences) using CellQuest Pro software.

### SiRNA transfection

Cells were seeded in 6 well plate (0.15 million per well) the day before transfection. Cells were transfected with 20 nM siRNA with Interferin transfection reagent (Polyplus) according to manufacturer’s protocol. A second transfection was performed after 24 h. Treatments were performed 48 h post-transfection. AHR siRNA was purchased from Dharmacon.

### Statistics

For qPCR and resazurin cell viability assays, statistical analysis was done by using two-tailed T test. Data are presented as means ± standard deviation (SD). A p-value of less than 0.05 was considered to be significant (p ≤ 0.05*, p ≤ 0.01**, p ≤ 0.001***).

## Supplementary information

Supplementary figures and tables.
