# Supplementary material for: Pifithrin-α alters p53 post-translational modifications pattern and differentially inhibits p53 target genes
Source: Sci Rep. 2020 Jan 23;10:1049. doi: 10.1038/s41598-020-58051-1 (PMC6978515; doi:10.1038/s41598-020-58051-1)
Supplement: Supplementary file 1 — Supplementary figures and tables. [file 41598_2020_58051_MOESM1_ESM.pdf]

Supplementary information

Pifithrin-α alters p53 post-translational modifications pattern and differentially inhibits p53 target genes

Jiawei Zhu, Madhurendra Singh, Galina Selivanova, Sylvain Peuget

Supplementary Table 1: qPCR primers

Gene	Forward primer (5'–3')	Reverse primer (5'–3')
**RPL13A**	CCTGGAGGAGAAGAGGAAAGAGA	TTGAGGACCTCTGTGTATTTGTCAA
**BBC3**	GACGACCTCAACGCACAGTA	CACCTAATTGGGCTCCATCT
**PMAIP1**	CATGAGGGGACTCCTTCAAA	TTCCATCTTCCGTTTCCAAG
**CDKN1A**	CTGTCACTGTCTTGTACCC	AGTGGTAGAAATCTGTCATGC
**MDM2**	CGTCTCCACACATCAGCACAA	CACTGTCCAACTTGACCCTCTTG
**PPM1D**	TTGTCAGAGCTGTGGAGGTG	CGATTCACCCCAGACTTGTT
**PIG3**	TAGCCGTGCACTTTGACAAG	ACTGGCCTTGTCTCTGCATT
**TP53INP1**	CAGAAATAGCCTTCGTCGCC	GTAATTGTACTGACGCGGGC
**SESN1**	ATTCGGCTGTGGAATCAGTC	TCCACACTGTGATTGCCATT
**RRM2B**	GGATCTCCCTCACTGGAACA	CGCTCCACCAAATTTTCATT
**TIGAR**	ATCCTGAAAGAAGCGGATCA	ACTGGCTGCTAATCCTGGAA
**ZMAT3**	GAATGAGCAATGTGGTCGAG	GGAACTGAAGGAGGCATCAC
**DRAM**	ATGGTCATCTCTGCCGTTTC	AAAGGCCACTGTCCATTCAC
**AHR**	ACTCCACTTCAGCCACCATC	ATGGGACTCGGCACAATAAA
**CYP1A1**	CAGCTGGATGAGAACGCCAAT	GTGTCAAACCCAGCTCCAAAG
**NRF2**	CGTTTGTAGATGACAATGAGG	AGAAGTTTCAGGTGACTGAG
**NQO1**	AGTATCCACAATAGCTGACG	TTTGTGGGTCTGTAGAAATG
**HO1**	CAACAAAGTGCAAGATTCTG	TGCATTCACATGGCATAAAG

Supplementary figure S1

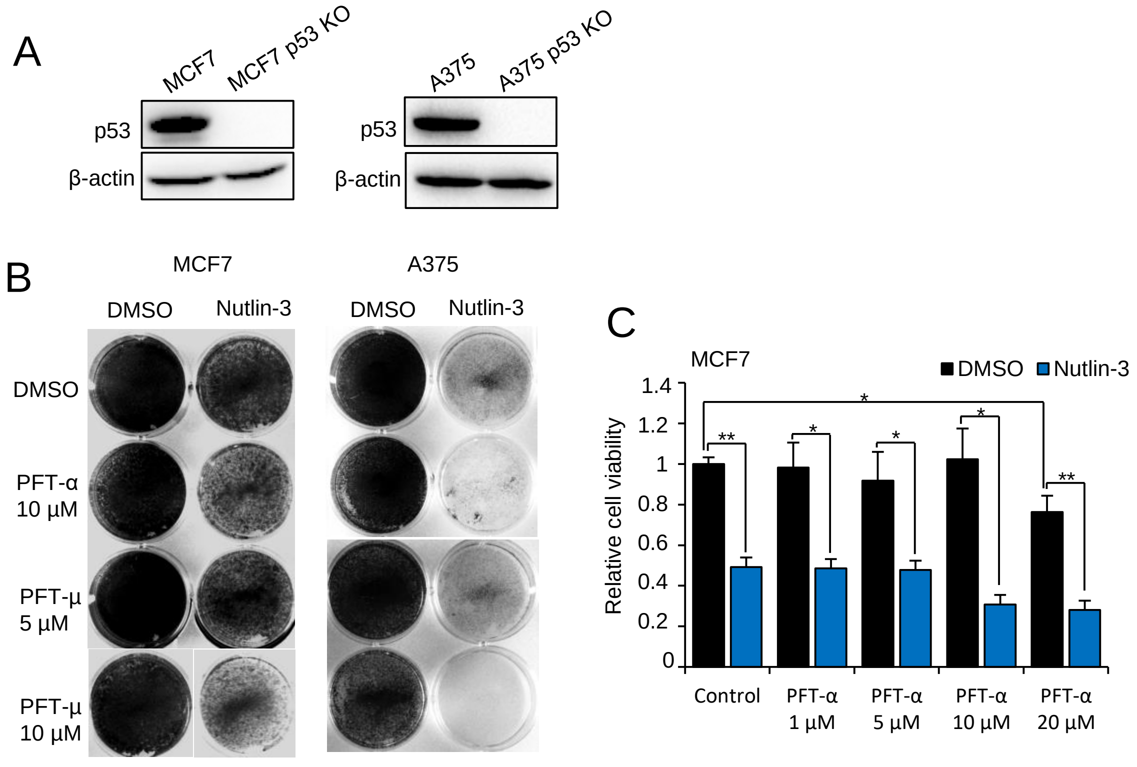

Supplementary Figure S1. A. Validation of p53KO in MCF7 and A375 cells by western blot. **B.** Crystal violet staining for cell viability upon 48h Nutlin-3 treatment (10 µM) in presence of PFT-α and PFT-µ in MCF7 and A375 cells. **C.** Resazurin cell viability assay of p53 wt MCF7 cells upon Nutlin-3 (10 µM) treatment in presence of PFT-α for 72h. The values are reported as relative to DMSO treatment group and represent the mean ± SD of three replicates.

Supplementary figure S2

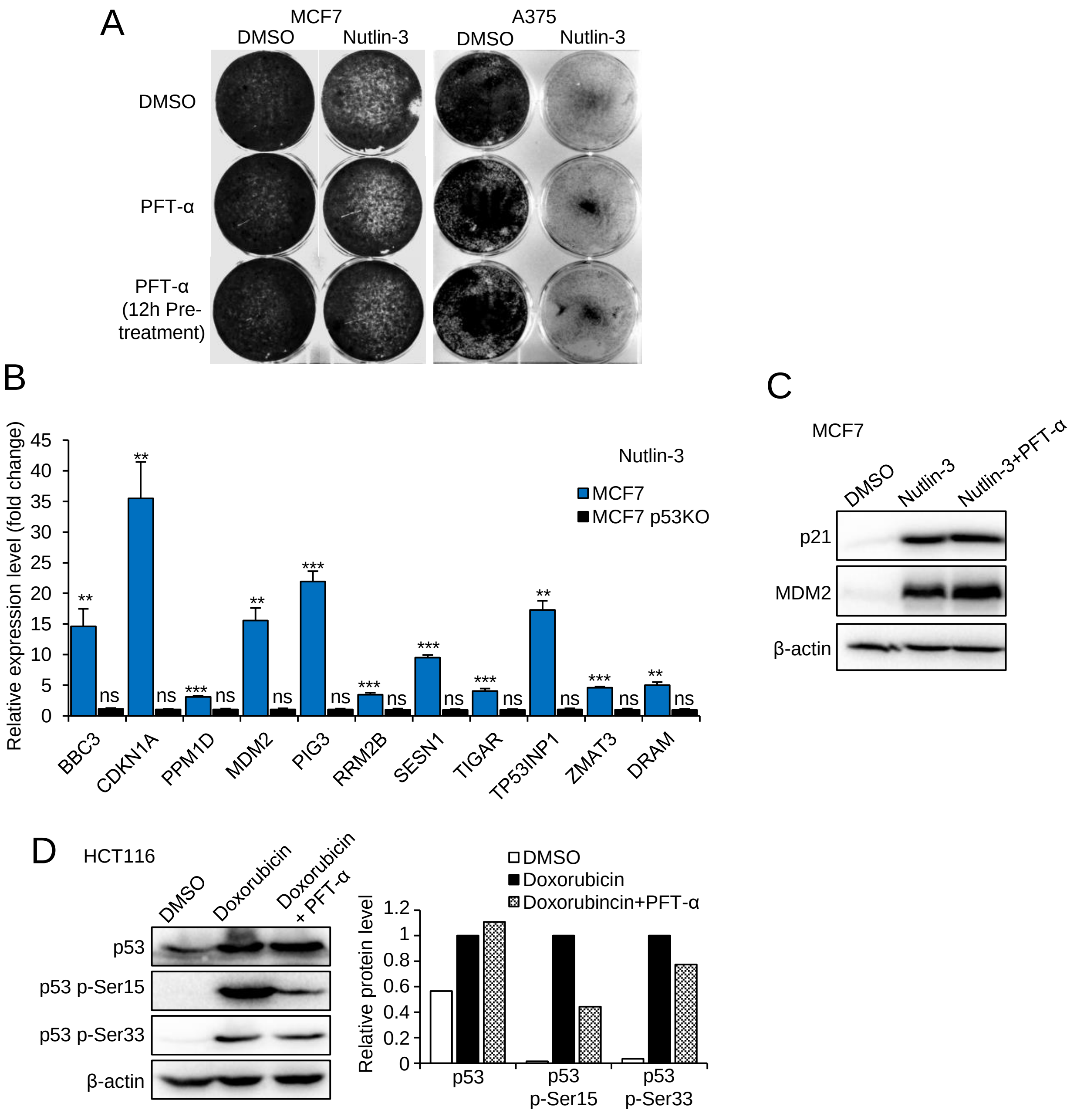

Supplementary Figure S2. A. Crystal violet staining for cell viability upon 48h Nutlin-3 treatment (10 µM) with pre- or co-treatment with PFT-α in MCF7 and A375 cells. **B.** qPCR for mRNA level on p53 transcriptional target genes in MCF7 and MCF7 p53KO cells with 8h 10 µM Nutlin-3 treatment. The values are reported as fold change relative to DMSO treatment group and represent the mean ± SD of three independent experiments performed in three replicates. **C.** Western blot for protein levels of p21 and MDM2 upon 8h Nutlin-3 treatment (10 µM) with or without PFT-α (20 µM, 12h pre-treatment) in MCF7 cells. **D.** Western blot to detect the protein level of p53, p53 p-Ser33 and p53 p-Ser15 upon 8h doxorubicin treatment (1 µM) with or without PFT-α (20 µM, 12h pre-treatment) in HCT116 cells. Densitometric analysis of the bands was performed using ImageJ software, the ratio of p53 p-Ser33/β-actin and p53 p-Ser15/β-actin for DMSO, doxorubicin and doxorubicin plus PFT-α treatment is quantified and then normalized with doxorubicin treatment group.

Supplementary figure S3

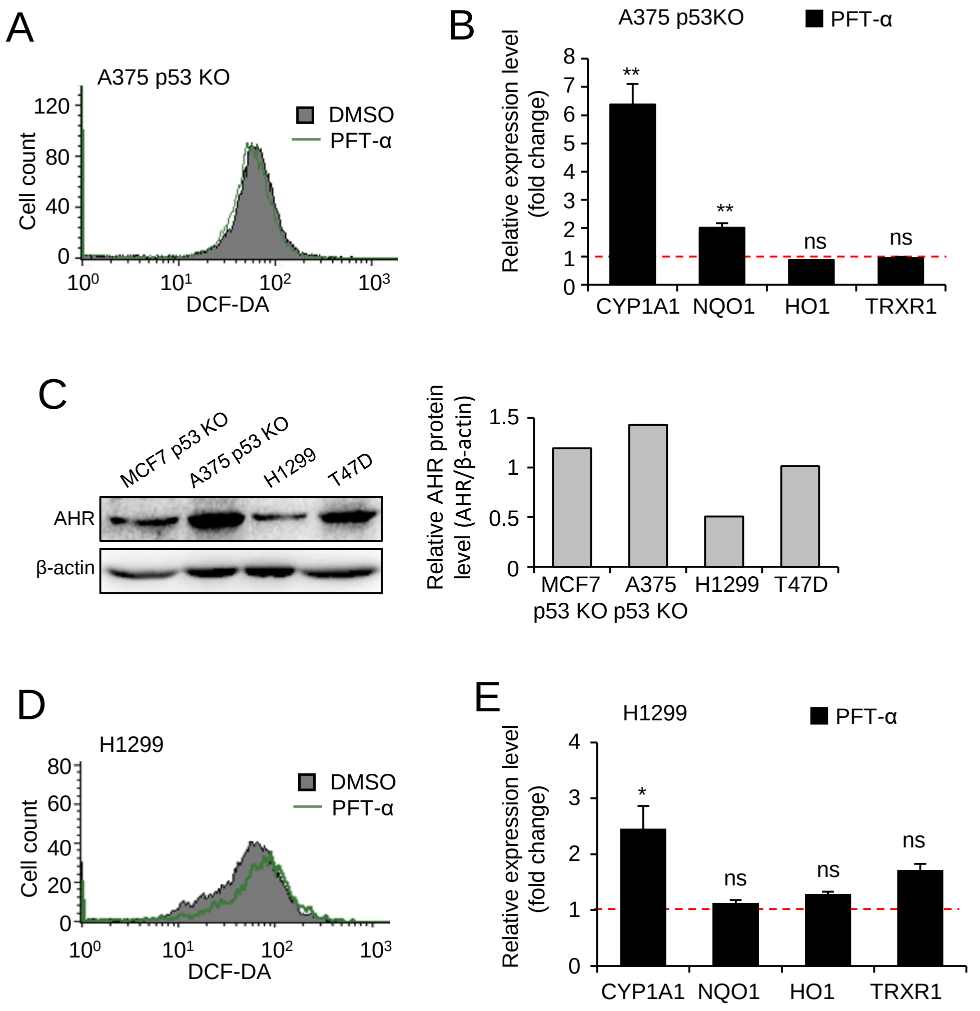

Supplementary Figure S3. A. DCF-DA staining of ROS levels upon 20h PFT-α treatment (20 µM) in A375 cells, detected using flow cytometry. **B.** qPCR to detect mRNA level of *CYP1A1, NQO1*, *HO1* and *TRXR1* upon 20h PFT-α treatment (20 µM) in A375 p53KO cells, normalized with DMSO treatment. **C.** Comparison of AHR protein level between MCF7 p53KO, A375 p53KO, H1299 and T47D cells analyzed by western blot. Right panel: densitometric quantification of the bands relative to actin. **D.** DCF-DA staining of ROS levels upon 20h PFT-α treatment (20 µM) in H1299 cells. **E.** qPCR to detect mRNA level of *CYP1A1, NQO1*, *HO1* and *TRXR1* upon 20h PFT-α treatment (20 µM) in H1299 cells, normalized to DMSO treatment.

Supplementary figure S4

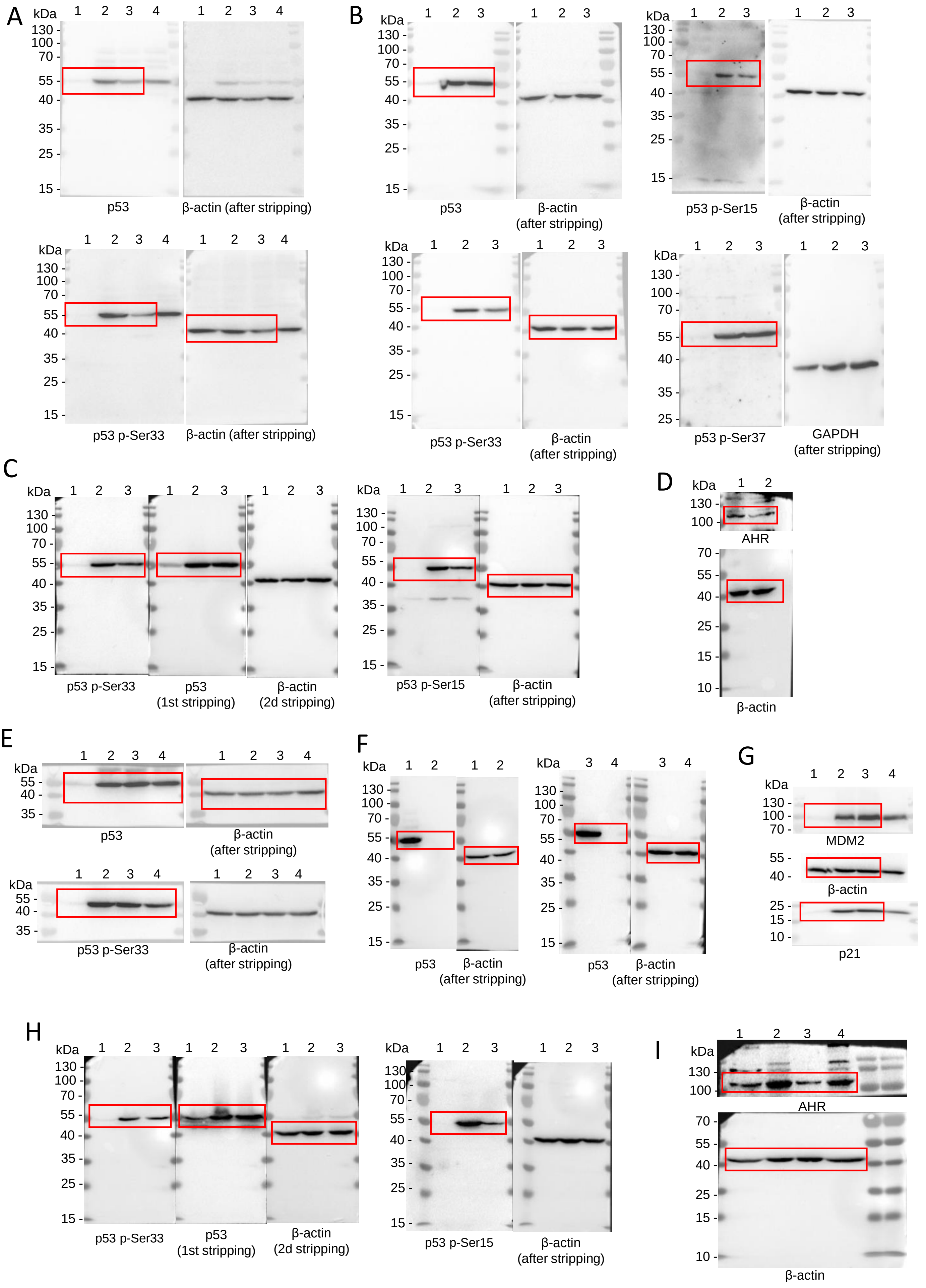

Supplementary Figure S4. Full-length, uncropped blots. Cropped parts presented in main figures are highlighted in red. For blots in A-C, the same membrane used for p53 or p53 PTMs antibody was stripped and revealed by anti-β-actin or anti-GAPDH. A representative β-actin blot is shown in main figures as loading control. **A.** Blots used in Fig. 2B. Lanes: 1) DMSO; 2) Nutlin-3; 3) Nutlin-3 + PFT-α. **B.** Blots used in Fig. 2C. Lanes: 1) DMSO; 2) Doxorubicin; 3) Doxorubicin + PFT-α. **C.** Blots used in Fig. 2D. Lanes: 1) DMSO; 2) Doxorubicin; 3) Doxorubicin + PFT-α. **D.** Blots used in Fig. 4B. The membrane was cut according to molecular weight ladder before incubation with the antibodies. Lanes: 1) Scramble; 2) SiAHR. **E.** Blots used in Fig. 4E. Membranes were cut according to molecular weight ladder before incubation with the antibodies. Lanes: 1) DMSO; 2) Nutlin-3; 3) Nutlin-3 + NAC; 4) Nutlin-3 + PFT-α. **F.** Blots used in Supplementary Fig. S1A. Lanes: 1) MCF7; 2) MCF7 p53KO; 3) A375; 4) A375 p53KO. **G.** Blots used in Supplementary Fig. S2C. The same membrane was cut according to molecular weight and revealed with specific antibodies against MDM2, p21 and β-actin. Lanes: 1) DMSO; 2) Nutlin-3; 3) Nutlin-3 + PFT-α. **H.** Blots used in Supplementary Fig. S2D. Lanes: 1) DMSO; 2) Doxorubicin; 3) Doxorubicin + PFT-α. **I.** Blots used in Supplementary Fig. S3C. The membrane was cut according to molecular weight ladder before incubation with the antibodies. Lanes: 1) MCF7 p53KO; 2) A375 p53KO; 3) H1299; 4) T47D.
